# Physical characterization and *in vivo* organ distribution of coated iron oxide nanoparticles

**DOI:** 10.1038/s41598-018-23317-2

**Published:** 2018-03-20

**Authors:** Anirudh Sharma, Christine Cornejo, Jana Mihalic, Alison Geyh, David E. Bordelon, Preethi Korangath, Fritz Westphal, Cordula Gruettner, Robert Ivkov

**Affiliations:** 1 Johns Hopkins University School of Medicine, Department of Radiation Oncology and Molecular Radiation Sciences, 1550 Orleans Street, CRB II, Baltimore, MD 21231 USA; 2 Micromod Partikeltechnologie GmbH, Friedrich-Barnewitz-St 4, D-18119 Rostock, Germany; 3 Johns Hopkins Bloomberg School of Public Health, Department of Environmental Health Sciences, Baltimore, MD 21205 USA; 4 Department of Mechanical Engineering, Whiting School of Engineering, Johns Hopkins University, Baltimore, 21218 USA USA; 5 Department of Oncology, Sidney Kimmel Comprehensive Cancer Centre, School of Medicine, Johns Hopkins University, Baltimore, MD 21231 USA; 6 Department of Materials Science and Engineering, Whiting School of Engineering, Johns Hopkins University, Baltimore, 21218 USA; 7 Institute for NanoBioTechnology, Whiting School of Engineering, Johns Hopkins University, Baltimore, 21218 USA

## Abstract

Citrate-stabilized iron oxide magnetic nanoparticles (MNPs) were coated with one of carboxymethyl dextran (CM-dextran), polyethylene glycol-polyethylene imine (PEG-PEI), methoxy-PEG-phosphate+rutin, or dextran. They were characterized for size, zeta potential, hysteresis heating in an alternating magnetic field, dynamic magnetic susceptibility, and examined for their distribution in mouse organs following intravenous delivery. Except for PEG-PEI-coated nanoparticles, all coated nanoparticles had a negative zeta potential at physiological pH. Nanoparticle sizing by dynamic light scattering revealed an increased nanoparticle hydrodynamic diameter upon coating. Magnetic hysteresis heating changed little with coating; however, the larger particles demonstrated significant shifts of the peak of complex magnetic susceptibility to lower frequency. 48 hours following intravenous injection of nanoparticles, mice were sacrificed and tissues were collected to measure iron concentration. Iron deposition from nanoparticles possessing a negative surface potential was observed to have highest accumulation in livers and spleens. In contrast, iron deposition from positively charged PEG-PEI-coated nanoparticles was observed to have highest concentration in lungs. These preliminary results suggest a complex interplay between nanoparticle size and charge determines organ distribution of systemically-delivered iron oxide magnetic nanoparticles.

## Introduction

Magnetic iron oxide nanoparticles (MNPs) have demonstrated utility in biomedical diagnosis and therapy because they display generally favorable biocompatibility and varied responsiveness to magnetic fields^1–10^. The search for nanoparticle constructs that preferentially accumulate in cancer tumors or cancer cells, after systemic delivery, remains an area of active research^11–15^. A successful strategy to develop selective targeting however requires knowledge of the relationship between nanoparticle structure and the resulting biologic activity between the physicochemical properties of nanoparticles and their impact on biological processes that affect the nanoparticle distribution throughout tissues and organs^16^. A precise working knowledge that reliably predicts such relationships remains a challenge for targeted nanomedicine^17^. Consideration of nanoparticle properties such as surface coating materials, size, zeta potential; and, the correlation of these with biodistribution *in vivo* remains a necessary and compelling area of investigation. In addition to relating the coating with physiological response, characterizing the magnetic properties is needed to determine if and how the coating affects the iron oxide responsiveness to magnetic fields.

When injected into circulating blood, nanoparticles encounter a complex fluid environment that includes living and non-living biological matter, and varying physical conditions. Interaction of nanoparticles with this dynamic environment can modify the initial particle surface to one having a molecular signature which produces specific interactions with host biology. Ultimately, though indirectly, the interactions of nanoparticles with the complex host environment can lead to final deposition in organs or tissues that depends upon the initial physical and chemical properties of the injected construct^16^. Therefore, a refined knowledge of these basic *in vivo* property-function correlations can benefit rational design of nanoparticle formulations for specific therapeutic applications.

We investigated, in pilot trials using healthy mice, the *in vivo* organ distribution of an iron-oxide magnetic nanoparticle (MNP) construct, previously used for *in vivo* cancer hyperthermia studies^18,19^. The surface coating of the nanoparticles was varied with polymer materials often used for this purpose^16,17,20–25^. Specifically, the nanoparticles were coated with carboxymethyl dextran (CM-dextran), polyethylene glycol-polyethylene imine (PEG-PEI), methoxy-PEG-phosphate + rutin, and dextran. Polymer coated particles were characterized for size, surface-charge or zeta potential, magnetic responsiveness, and heat generation. Following intravenous delivery (48 hours), animals were sacrificed and tissues were collected to measure iron concentrations with inductively coupled-plasma mass spectrometry (ICP-MS) in livers, spleens, and lungs. Recovered iron content, a surrogate measure of nanoparticle deposition, was highest in livers of mice injected with CM-dextran nanoparticles. On the other hand, iron content was highest in lungs of mice injected with PEG-PEI coated nanoparticles. These pilot results suggest various complex biological mechanisms engage, depending upon both size and surface charge of injected nanoparticles.

## Results and Discussion

Table 1 contains a summary of MNP physical data. For each coating, nanoparticle size [*Z*(Avg)] and polydispersity index (P.I.) were evaluated in water and in PBS separately at an iron concentration of 0.4 mg/ml using dynamic light scattering. Iron concentration for each lot was determined using spectrophotometry (see Methods in Supporting Information).

**Table 1:** Physical properties of nanoparticle suspensions.

Coating (Lot)	c(Fe) [mg/ml]	Z(Avg) [nm] in H2O	P.I. in H2O	Z(Avg) [nm] in PBS	P.I. in PBS
Uncoated (Citrate-stabilized) [90805]	35.3	77	0.179	1206	1.000
CM-dextran [5170945]	15.7	240	0.211	211	0.180
PEG-PEI [5100945]	30.7	230	0.162	219	0.202
Methoxy-PEG-phosphate + Rutin [5230945]	21.2	79	0.145	296	0.324
Dextran[5110945]	10.9	73	0.147	74	0.106

The precursor citrate-stabilized iron oxide particles demonstrated evidence of coagulation in PBS displaying significantly increased hydrodynamic diameter (>15-fold), and formation of precipitates. To a lesser extent, the methoxy-PEG-phosphate + rutin coated nanoparticles exhibited similar instability in PBS. These results are consistent with previous observations of charge-stabilized nanoparticle suspensions when in physiologic solutions or biologic media^26–29^.

As expected, particles coated with high molecular weight polymers were stable in either water or PBS (Table 1). The measured mean hydrodynamic diameter of dextran-coated nanoparticles remained constant in either water or PBS demonstrating relative insensitivity to changes of solvent conditions within physiologic parameters. It is worth noting that, while stable in either water or PBS, the measured mean hydrodynamic diameters of the CM-dextran and PEG-PEI coated nanoparticles decreased slightly when in PBS vs water. The presence of available carboxyl or amine groups on the polymer chain is likely responsible for these differences because their presence can introduce complex responses to changes of solution pH or ionic strength^26–29^. Indeed, this complexity has been proposed as a potential advantage for some nanoparticle formulations to achieve specific therapeutic objectives within the environment of tumors which typically exhibit low pH^30,31^.

The basic framework for understanding the stability of charged colloids is the Derjaguin-Landau-Verwey-Overbeek (DLVO) model^28,29,32^, which identifies colloid stability to be the consequence of a balance between attractive and repulsive forces among charged colloids, and their interactions with solvent ions. Increasing ionic strength of the solvent (or changing pH), can have the effect to change the surface potential and compress the counter-ion cloud (i.e. electric double layer) leading to a reduced zeta potential and hydrodynamic diameter. On the other hand, a highly compressed double layer can effectively reduce the repulsive forces between MNPs, giving rise to agglomeration and increased hydrodynamic diameter. In the extreme limit, colloid aggregates become too large to be supported by Brownian motion and the particles begin to flocculate. We conclude that both citrate stabilized precursor and methoxy-PEG-phosphate + rutin coated particles were unstable in PBS because ionic stabilization is inadequate to maintain colloid integrity in higher ionic strength solvents^26,33,34^. Dextran, carboxymethyl dextran and PEG-PEI, on the other hand are long-chain high MW polymers that provide additional steric stabilization making these suspensions less sensitive to changing ionic strength within physiologic parameters. These observations are generally consistent with prior work, leading to the prevalence of these polymers as nanoparticle coating agents for many biomedical applications^26,33^.

**Figure 1:**
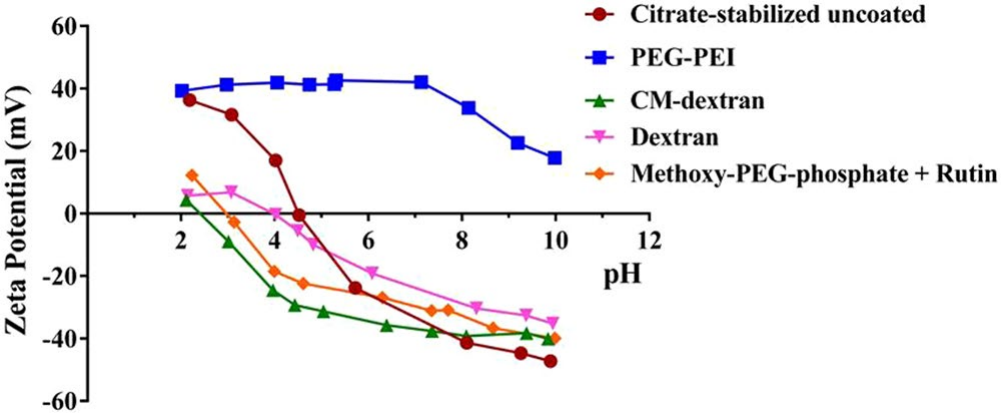
Measured pH-dependent zeta potential of magnetic iron oxide nanoparticles (MNPs) coated with citrate (red), PEG-PEI (blue), CM-dextran (green), dextran (magenta), and methoxy-PEG-phosphate + rutin (amber) in water. All MNP constructs displayed a negative surface charge at pH 7, except PEG-PEI MNPs.

The observed slight reduction of mean hydrodynamic diameter displayed by nanoparticles coated with the carboxy- or amine-containing polymers is likely due to the increased sensitivity of the colloid surface potential to H^+^. Interestingly, CM-dextran- and PEG-PEI-coated MNPs displayed relatively high values of zeta potential at pH 7 (Fig. 1). In these cases, the ionic strength of PBS may contract the double layer, effectively reducing the thickness of the polymer layer (i.e. reducing radius of gyration and expelling solvent). A collapse of the polymer-double layer and dominance of attractive forces was likely prevented by polymer steric stabilization (monomer-monomer exclusion)^26,32^.

The pH-dependent zeta potential measured for each MNP is shown in Fig. 1. Citrate-stabilized precursor MNPs displayed a high, positive zeta potential at low pH (<4), with point of zero charge (PZC) occurring at approximately 4.5. All other coated nanoparticle samples exhibited positive zeta potential at low pH, though only citrate- and PEG-PEI-coated nanoparticles exhibited high (measured maximum) zeta potential values>+20 mV at pH < 3. All particles, except PEG-PEI displayed PZC at pH ~4. The PZC of the CM-dextran-nanoparticles occurred at pH ~2.4, and zeta potential below PZC was <5 mV. At pH above the PZC, the measured zeta potential of all particles, except PEG-PEI was negative. PEG-PEI-nanoparticles did not display PZC to pH 10, and at a physiologic pH 7.4, the measured zeta potential was ~+40 mV. By contrast the measured zeta potential at pH 7.4 for CM-dextran particles was −38 mV. The measured zeta potential of all other nanoparticles at pH 7.4 was similarly negative, although with slightly lower magnitude than the CM-dextran-nanoparticles. It is interesting to compare the sizes and surface potentials of the PEG-PEI with those of CM-dextran nanoparticles. Both exhibit high values of zeta potential at physiologic pH, with one having positive (PEG-PEI) sign and the other negative (CM-dextran); and, both these coated MNPs have comparable measured mean hydrodynamic diameter (Table 1).

The magnetic susceptibility of MNPs is given by $\tilde{\chi }$ = *χ*′ + *jχ*″ where, χ′(ω) = *χ*_0_/[1 + (*ωτ*)^2^] and *χ*″(*ω*) = *χ*_0_ *ωτ* /[1 + (*ωτ*)^2^]. *χ*_0_ is the static (DC) magnetic susceptibility, *ω* is the frequency of the applied magnetic field and *τ* is the relaxation time of the MNPs^35,36^. When suspended in a fluid and exposed to an external field, the magnetic moments of the MNPs will align with the external magnetic field vector, giving rise to the measured value of magnetization. Upon removal of the external field, randomization of the individual magnetic moments leads to decay of magnetization, or relaxation. Suspensions containing free MNPs realize relaxation via two possible physical processes, Brownian and Néel. Néel relaxation can be described as a thermal relaxation process of the magnetic moment against an effective anisotropy energy barrier, which originates from a combination of anisotropies such as magneto-crystalline, shape, surface, etc. in the MNP. Brownian relaxation results from physical rotations of the nanoparticles and is directly associated with the magnetic torque on the MNPs against the viscosity of the suspending medium and thermal fluctuations^35,36^. Depending upon the magnetic and physical characteristics of the nanoparticles, suspending medium and experimental conditions, Brownian relaxation is usually observed to be much slower than Néel relaxation. For magnetically blocked nanoparticles, where thermal energy is insufficient to overcome the anisotropy barrier, but the MNPs are free to move physically, relaxation may be dominated by Brownian processes. In the suspending medium the Brownian relaxation time can be described by the relation^35–38^

1 $${\iota }_{B}=3\eta {V}_{H}/{K}_{B}T$$

where *V*_*H*_ is the hydrodynamic volume of the particle, *η* is the dynamic viscosity of the liquid, *K*_B_ is the Boltzmann constant and *T* is absolute temperature.

Magnetic single-domain nanoparticles exhibiting faster internal relaxation, i.e. unblocked or ‘quasi’-blocked at the experimental conditions will also display a Néel contribution to relaxation. Typically, for single-domain particles in null external field, the Néel relaxation time is taken to be^35,36^:

2 $${\tau }_{N}={\tau }_{A}(\frac{\sqrt{\pi }}{2})(\sqrt{\frac{{k}_{B}T}{K{V}_{M}}}){e}^{K{V}_{M}/{k}_{B}T}$$

where *τ*_*A*_ is the attempt frequency (~10^−9^ sec^−1^), *V*_*M*_ is the magnetic volume, and *K* is the anisotropy constant, which includes all contributions to the anisotropy. This functional form for Néel relaxation time only applies for the special case of null magnetic field^35^. With application of an external magnetic field, complex ‘multi-domain’ internal magnetic structures, and contributions from dipole interactions in suspension or in aggregates will inevitably modify the relaxation time. In many cases both relaxation mechanisms can operate for a given MNP, yielding an effective relaxation time of *τ* = (*τ*_*n*_ × *τ*_*b*)_/(*τ*_*n*_ + *τ*_*b*_)^35–38^. Generally, the frequency of Brownian relaxation of nanoparticles suspended in water is ~1 s^−1^ to 10^4^ s^−1^ ^36^. Néel relaxation times can range from 10^−9^ s to 10^9^ s, depending upon the anisotropy energy and volume of nanoparticle, and experimental conditions^36–38^.

For dextran coated MNPs, the effective anisotropy *K*_*eff*_ has been measured to be 0.51 J/kg Fe (3.66 × 10^3^ J/m^3^) and a magnetic volume *V*_*m*_, that is a magnetic spherical domain of about 36 nm diameter (assuming single domain sphere and neglecting magnetic shell)^19^. Using the dynamic viscosity of water, *η* = 9 × 10^−4^ Pa.s, Boltzmann constant, *k*_*B*_ = 1.38 × 10^−23^ m^2^·kg·s^−1^·K^−1^, temperature, *T* = 300 K, and a hard sphere hydrodynamic volume *V*_*H*_, of 77 nm; we obtain values of *τ*_*B*_ and *τ*_*N*_ to be 1.5 × 10^−4^ s and 4.5 × 10^−1^ s, respectively.

**Figure 2:**
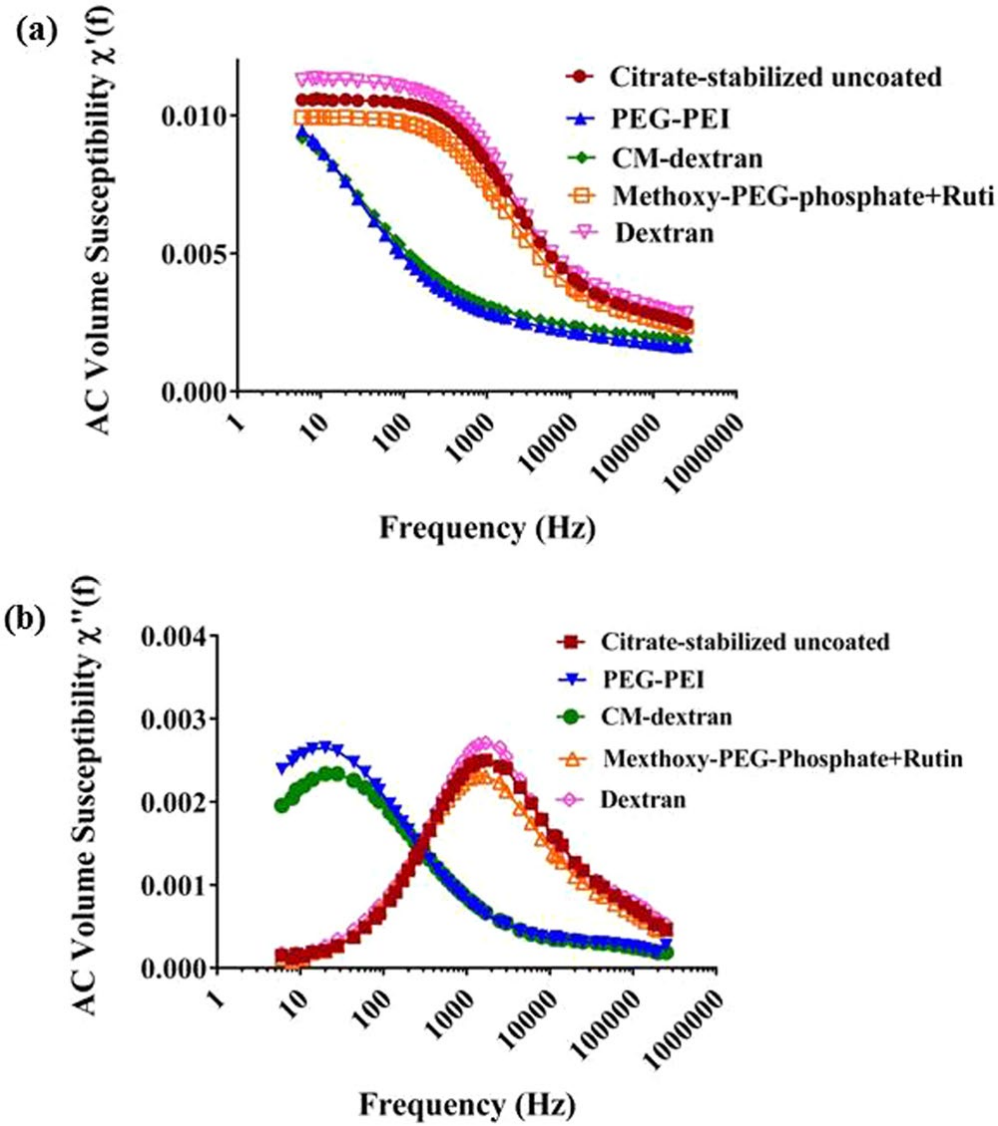
(**a**) The real component of AC volume susceptibility, *χ*′, measured from coated MNPs vs frequency suppressed in PEG-PEI and CM-dextran MNPs at frequencies 100–1000 s^−1^, a result of increased hydrodynamic volume and reduce mobility. (**b**) The imaginary component of AC susceptibility, *χ*″, measured from coated MNPs vs frequency displays a peak which shifted to lower frequencies when MNPs were coated with CM-dextran or PEG-PEI to yield particles having larger measured hydrodynamic diameter (~200 nm) compared to their uncoated precursor MNPs (~80 nm). Other coated MNPs having sizes similar to the citrate-stabilized precursor MNPs had both *χ*′ and *χ*″ that remained close to original. MNP concentration was 1 ± 0.05 mg Fe/ml for all measurements.

A peak in the out-of-phase component, *χ*″, corresponds to the alternating magnetic field (AMF) frequency at which optimal phase lag occurs^36,38^ i.e., *ωτ* = 1. The uncoated citrate stabilized MNPs exhibit a broad peak in the complex (imaginary component) susceptibility, *χ*″, at ~1.5 to 2 × 10^3^ s^−1^ as shown in Fig. 2(b) which is in the frequency range where Brownian relaxation is expected to dominate according to calculations shown above. Using the expression for Brownian relaxation and measured hydrodynamic diameter of MNPs, the expected Brownian relaxation peak for mobile non-interacting MNPs was estimated to be ~1 × 10^3^ s^−1^, which approximately corresponds with the observed susceptibility peak displayed in Fig. 2(b) (~1.5-2 × 10^3^ s^−1^). While the calculated peak is within the same order of magnitude as the observed peak of the complex susceptibility, the observed differences suggest the assumptions that the MNPs are monodisperse, non-interacting hard spheres may be incorrect. Deviations of MNP shape from a perfect sphere and dipolar interactions among MNPs producing collective magnetic behavior can also explain differences between measured and calculated values^39^. Polydispersity in particle shape introduces complex deviations in Brownian relaxation calculations which assume a spherical object. Medium viscosity is also a dominant contribution to Brownian relaxation processes that interacts directly with particle volume. Polydisperse particle size, thus introduces deviations with increased relaxation times (slower) for larger particles and correspondingly decreased relaxation times (faster) for smaller particles, effectively broadening the peak^40^. A distribution *p*(*τ*) of relaxation times can be introduced to the Debye model to account for size polydispersity:

3 $$\tilde{\chi }(\omega )={\chi }_{0}{\int }_{0}^{\infty }p(\tau )\frac{1}{1+j\omega \tau }d\tau .$$

Without further assumptions, a numerical inversion can be used to calculate *p*(*τ*) from AC susceptibility (ACS) data^41^ (Supplementary Information). The peak observed at 1 × 10^−4^ s in Figure S2, matches approximately the calculated value for Brownian relaxation time. Immobilization of these MNPs in agarose suppresses this major peak, thus providing evidence that a significant contributing process to this peak is Brownian relaxation.

On the other hand, a second peak is observed in the numerical inversion of ACS data that occurs at ~7 × 10^−7^ s, and persists when the MNPs are immobilized in agarose gel (Figure S2). Even with a broad size distribution of the hydrodynamic diameter, the relaxation time distribution must include relaxation times below 7 × 10^−7^ s (or a resonance frequency of 2.5 × 10^5^ s^−1^) to provide an acceptable fit to ACS data (Figures S1 and S2). This suggests that a faster relaxation process also contributes to the measured ACS values. We suspect the origin of this peak includes Néel relaxation; however, while the second peak in Figure S2 (~3 × 10^−6^ s) is significant, it is close to the upper limit of the susceptometer (2.5 × 10^5^ s^−1^), making detailed comparisons unreliable.

For larger MNPs (PEG-PEI and CM-dextran with d ~230–240 nm), the estimated Brownian relaxation is ~36 s^−1^. The measured *χ*″ peak and therefore, Brownian relaxation is suppressed at 2 × 10^3^ s^−1^ and shifted to lower frequency (~30 s^−1^). This can be attributed to reduced mobility arising from the increased hydrodynamic diameter with CM-dextran or PEG-PEI coating. Both complex, *χ*″, and real, *χ*′, components of susceptibility are suppressed and shifted to lower frequencies for these MNPs at 2 × 10^3^ s^−1^ at the measured frequency range 100–1000 s^−1^. Uncoated, dextran-coated, and methoxy-PEG-phosphate + rutin-coated MNPs displayed similar susceptibilities owing to comparable diameters of these MNPs (Fig. 2).

**Figure 3:**
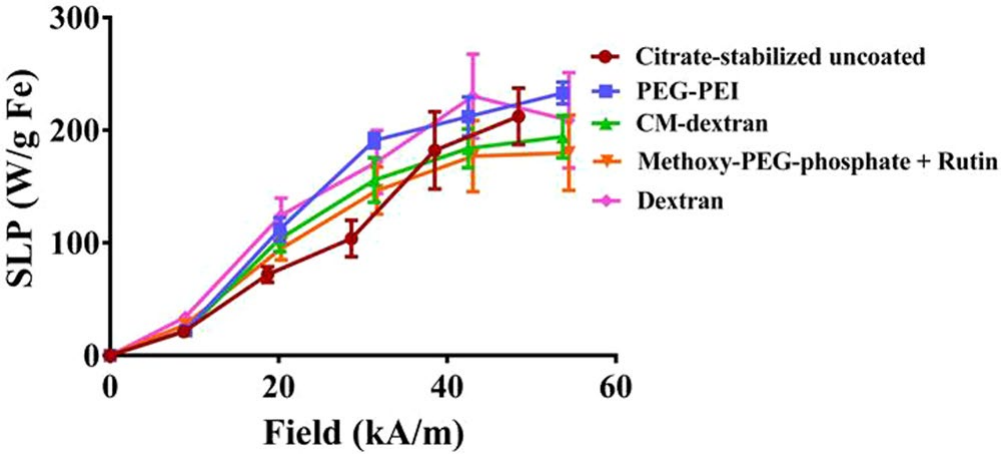
Specific loss power (SLP), reported as W/g Fe vs applied magnetic field was measured at fixed frequency (1.50 ± 10 × 10^5^ s^−1^ kHz) demonstrates surface coating has a minimal impact on the SLP (variations < 10%). The SLP values reported are the mean of all values obtained from a heating measurement that satisfied the (quasi)-adiabatic criteria as described in ref.^51^ (see text for details). Error bars are the standard deviation of mean values.

The MNPs were exposed to a time-varying or alternating magnetic field (AMF) having amplitude up to 54 kA/m and frequency 1.5 × 10^5^ s^−1^ to measure heat production by the MNPs. It is worth noting that the magnetic field to which the MNPs were exposed for heating experiments exceeds, in both magnitude and frequency, the AMF used for ACS measurements (see Methods). Heating rates measured from the coated MNPs were comparable to those of the uncoated citrate-stabilized MNPs (Fig. 3). The precursor iron oxide nanoparticles exhibited a non-linear amplitude-dependent specific loss power (SLP) with applied AMF. It has been previously reported that this non-linear response likely arises from intra-core magnetic domain structure and complex coupling among magnetic domains within the iron oxide cores^19^, and the heating was relatively unaffected by surface coating.

**Figure 4:**
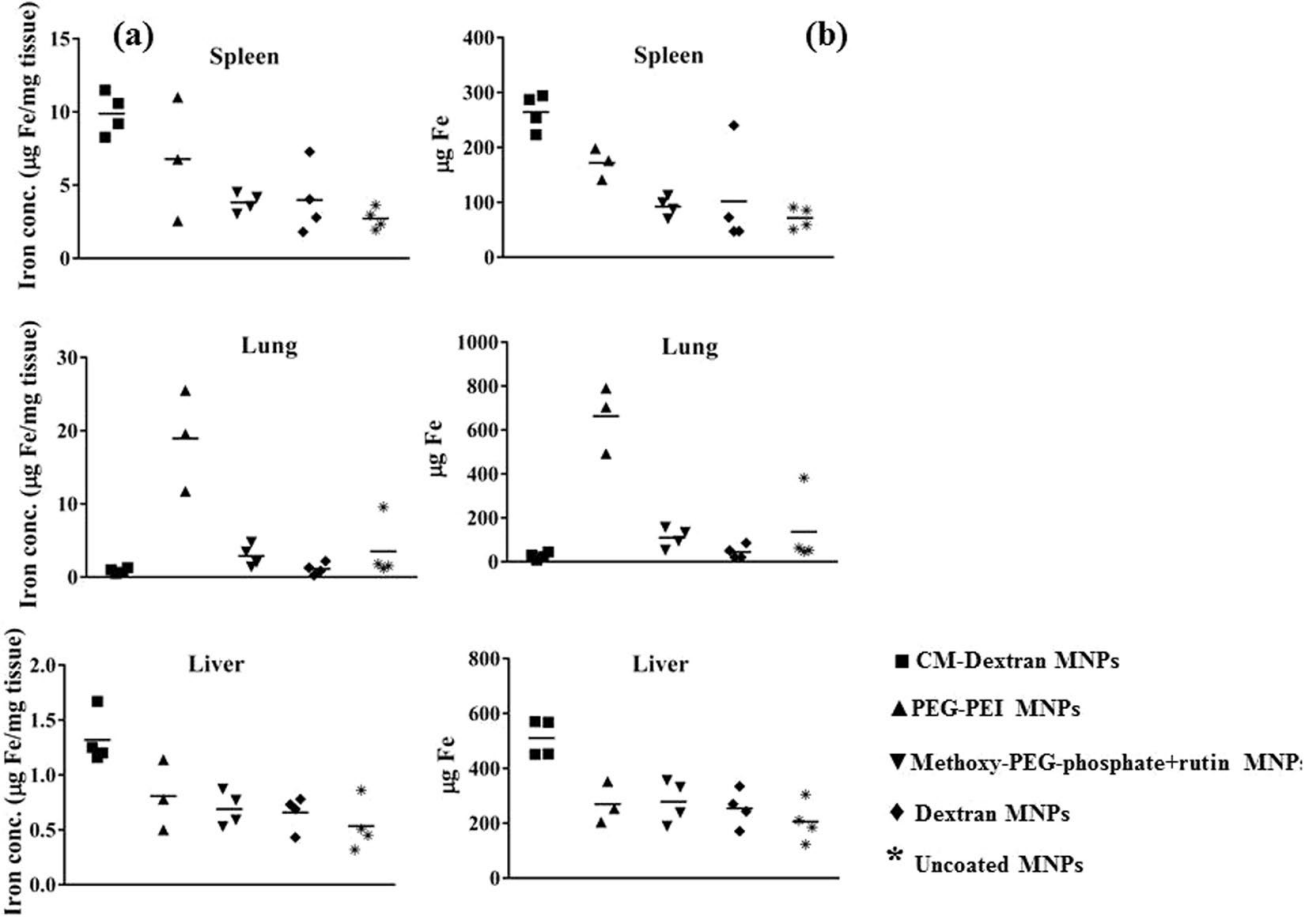
(**a**) Concentrations (μg/mg tissue) of Fe recovered from spleens, lungs, and livers of mice injected with MNP suspensions. Iron measurements were obtained using inductively-coupled plasma mass spectrometry (ICP-MS) from processed tissues that were collected 48 hours after intravenous injection. Symbols display individual data obtained from a single mouse and median values within a group are indicated by a horizontal line. (**b**) Plot showing total recovered Fe (total mass in μg) measured by ICP-MS from tissues as described in (**a**). (■) represents CM-dextran MNP, (▲) represents PEG-PEI MNP, (▼) represents methoxy-PEG-phosphate + rutin MNP, (♦) represents dextran MNP, (*) represents uncoated MNP.

**Figure 5:**
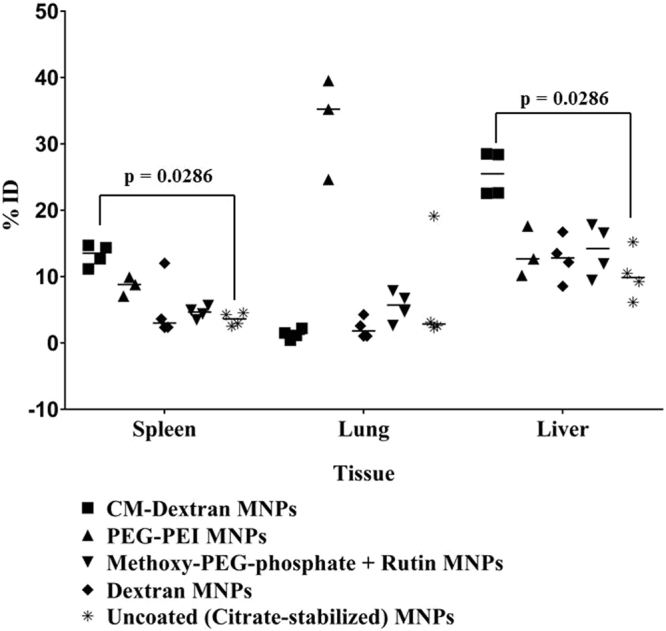
Scatter plot showing calculated percent of injected dose (ID) of iron recovered from livers, spleens and lungs of mice injected with coated MNPs. N = 4 mice for each group, except PEG-PEI MNPs, for which N = 3. (■) represents CM-dextran MNP, (▲) represents PEG-PEI MNP, (▼) represents methoxy-PEG-phosphate + rutin MNP, (♦) represents dextran MNP and (*) represents uncoated MNPs. Statistical analysis of data was performed using non-parametric Mann-Whitney test demonstrating significant differences in deposition in livers and spleens between CM-dextran MNPs and uncoated precursor (citrate-stabilized) MNPs (p = 0.0286). Comparison of iron concentrations measured in lungs of mice injected with PEG-PEI MNPs with those injected with CM-dextran, or with uncoated MNPs yields p = 0.057.

To determine if the coating material influences organ deposition, healthy male athymic nude mice were injected intravenously with MNP suspensions. Generally, injections of MNPs were well tolerated within the observed time. The exception was one death out of four mice injected with PEG-PEI coated MNPs. 24 and 48 hours later, blood samples were collected for iron content analysis, and at 48 hours all mice were sacrificed and livers, lungs and spleens were harvested. Iron content in blood and tissues was measured with ICP-MS to determine nanoparticle content in tissues. At both measured time points, blood iron content was similar to controls (data not shown), suggesting that nanoparticle clearance from blood circulation occurred within 24 hrs. Results of organ iron analysis are provided in Fig. 4(a) and (b). Most interesting is a comparison of recovered iron measured in organs extracted from mice injected with PEG-PEI and CM-dextran MNPs. Coating the precursor nanoparticles with these materials produced nanoparticles having similar size and iron oxide composition (Table 1), and measured zeta potential having similar magnitude but opposite sign (Fig. 1). Measured iron was highest in spleens and livers of mice injected with CM-dextran MNPs, but it was highest in lungs of mice injected with PEG-PEI MNPs; similar to previously reported results for nanoparticles having comparable size and surface charged density^42–49^. When normalized with respect to initial injected dose of iron (Fig. 5), approximately 34% of the injected iron dose of PEG-PEI MNPs accumulated in the lungs compared to ~1% for CM-dextran (p = 0.057, borderline significance). The borderline statistical significance of the comparison is a consequence of the single death in this study group (N = 3), compared to other treatment groups (N = 4). Livers and spleens of mice injected with CM-dextran MNPs showed much higher accumulation of iron (26% and 13%, respectively) compared to mice injected with PEG-PEI MNPs (14% and 11%, respectively). Iron recovered from mice injected with uncoated, dextran, or methoxy-PEG-phosphate + rutin-coated MNPs yielded similar values with the maximum accumulating in the livers. The death following PEG-PEI MNP exposure warranted a more complete examination of potential toxicity arising from the use of PEG-PEI to coat magnetic iron oxide nanoparticles^42,43^.

To explore the nature of biological factors associated with accumulation of PEG-PEI MNPs in lungs, we conducted a follow-up study. In this latter study, only lungs were harvested and prepared for analysis by histopathology. The additional cohorts of mice were injected intravenously with PEG-PEI MNPs (n = 5) and CM-dextran (n = 5) MNPs, summarized in Table 2. Initially, intravenous injections were given at a dose of 2 mg Fe for both PEG-PEI and CM-dextran MNPs. Within 24 hours after injection, however all mice in PEG-PEI cohort died, indicating severe toxicity associated with this construct. Post-mortem histopathology of lungs indicates acute lung tissue toxicity (Supplementary Materials Figures S3).

**Table 2:** Physical properties of nanoparticle suspensions used for Histology sub-group.

Coating	c(Fe) [mg/ml]	Z(Ave) [nm]	P.I.	Z(Ave) [nm] in PBS	P.I. in PBS	Zeta potential (mV)
(Lot)		in H2O	in H2O			at pH 7
CM-dextran [0451745-001]	22.8	171	0.153	272	0.405	−38
PEG-PEI [0461745-001]	23.4	166.6	0.115	136.4	0.123	28

We repeated the trial with another cohort of mice (n = 4) using a lower dose of PEG-PEI MNPs, 1 mg Fe. All mice in this cohort and CM-dextran cohort survived to the endpoint of the study (48 hours), and lungs from all mice were collected and prepared for histopathology. Although no acute toxicity was observed in either PEG-PEI 1 mg or CM-dextran injected mice, infiltration of immune cells was observed throughout lungs to varying degrees in both cohorts. (Supplementary Materials Figure S4.

**Figure 6:**
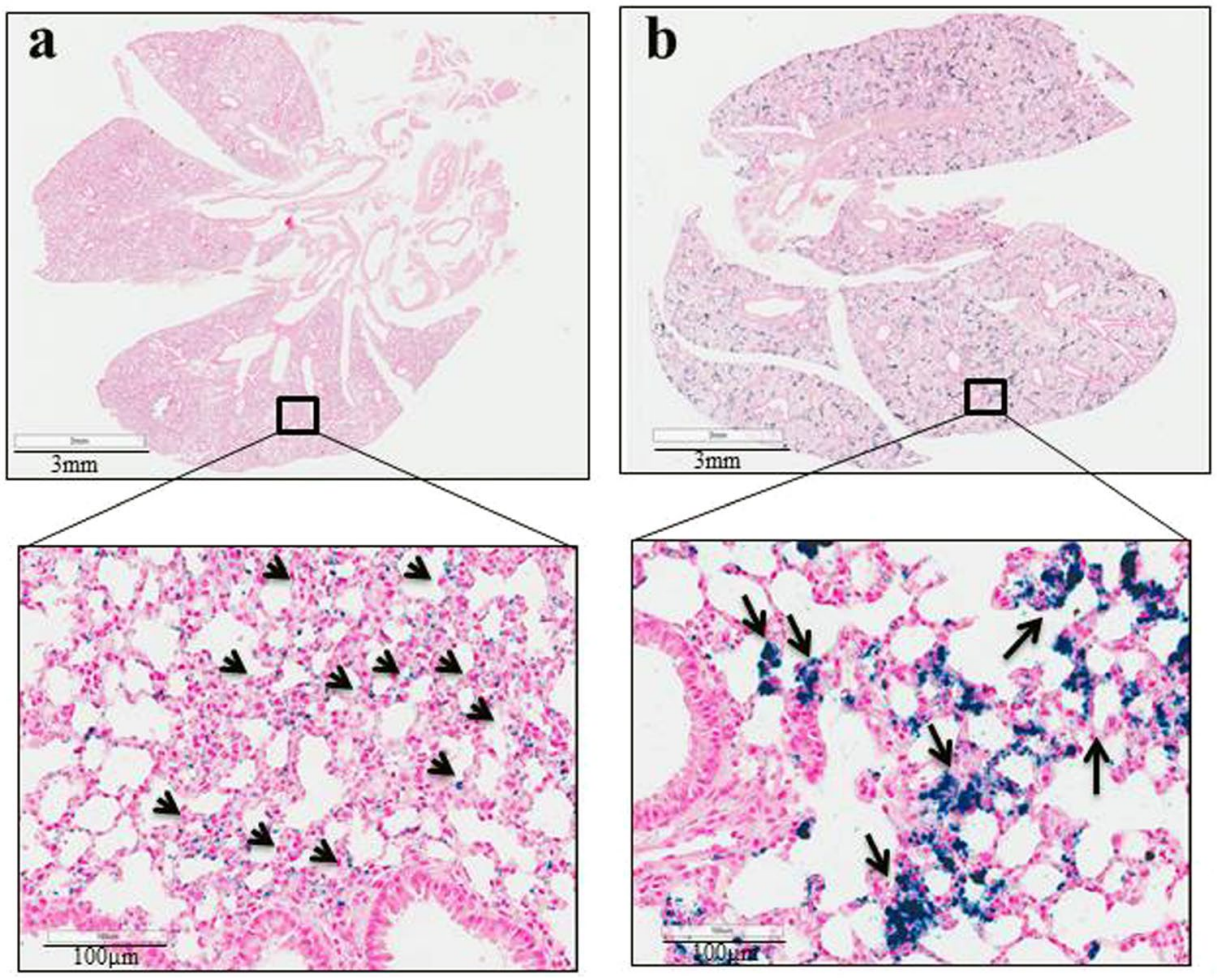
Representative images of Prussian blue stained lung sections of animals treated with MNPs shows distinct patterns of iron accumulation in lungs depending upon particle coating and dose. (**a**) Lung tissue section from mouse injected with PEG-PEI MNPs (1 mg Fe) showing Prussian blue positive staining appears to have a dispersed pattern and concentrations of blue coloring (nanoparticle rich regions) correlate with alveolar epithelial cells, indicating nanoparticle accumulation in these cells. (**b**) Lung of mouse injected with CM-dextran MNPs showing a more intense clustering of particles within the interstitial spaces, a distinctly different manifestation than with PEG-PEI MNP distribution. The lower panel of figures provides a 20× magnified view of the boxed region in upper panels.

As indicated by Prussian blue staining, PEG-PEI MNP localization appears to be concentrated within or near lung epithelial cells (Fig. 6a), whereas CM-dextran MNP localization was concentrated within interstitial spaces (Fig. 6b). Staining for mouse macrophages and monocytes revealed no clear association between either nanoparticle construct with lung macrophages or monocytes (Supplementary Materials Fig. S5). This is surprising considering macrophages are considered the principal immune cell population that interacts with nanoparticles to clear them from blood circulation^24,44–49^.

In summary, we report results of characterization of magnetic iron oxide nanoparticles coated with several biocompatible polymers. The choice of coating material determined the physical properties of the nanoparticles in solution depending on pH and solvent matrix. Measured hydrodynamic diameter and surface charge density (i.e. zeta potential) among the nanoparticles were relatively similar except PEG-PEI and CM-dextran coated nanoparticles. Other than size-dependent changes to colloid dynamics, we determined that no remarkable changes occurred to time-dependent magnetic processes following coating. The PEG-PEI and CM-dextran nanoparticles presented an interesting pair for comparing effects of nanoparticle charge (similar in magnitude but opposite sign) on organ distribution following systemic (intravenous) exposure. Contrary to expectations and unlike their negatively charged counterparts, the positively charged PEG-PEI nanoparticles proved more toxic and accumulated preferentially in lungs of mice 48 hours after injection. Negatively charged nanoparticles were found to have preferentially localized in livers. In a follow up study, histopathology in lungs revealed that the PEG-PEI nanoparticles co-localized near or within lung epithelial cells. No evidence was found of nanoparticle localization with macrophages. From these results, we conclude that multiple immune and epithelial cells interact with nanoparticle constructs. The nature of *in vivo* cell interactions, across multiple cell and tissue types, with nanoparticles merits closer investigation with quantitative tools and histopathology.

## Materials and Methods

All reagents were analytical grade purchased from SIGMA-ALDRICH Chemie GmbH (Taufkirchen, Germany) unless specified otherwise. 0.22 µm filtered reverse osmosis water was used in all preparations of coated nanoparticles. Citrate-stabilized iron oxide magnetic nanoparticles (MNPs) were produced by high-gravity controlled precipitation (HGCP) (NanoMaterials Technology Ltd., Singapore) from aqueous solutions of precursor FeCl_2_ and NH_4_OH. Details for MNP synthesis are described elsewhere^46^. No further modifications of nanoparticles were performed prior to coating.

### Coating MNPs

Dextran coating of the MNPs was achieved by dissolving 18 g of dextran (M.W. = 40,000 g/ml; Carl Roth GmbH, Karlsruhe, Germany) in 60 ml of water in a glass beaker, and heating to 60 °C. 45 ml of citrate MNP suspension (solid content 50 mg/ml) was diluted with 70 ml of water in a 500 ml glass flask. The citrate MNP suspension was added to the reservoir of a microfluidizer (M-110EH, Microfluidics, Newton, MA, USA), and was circulated for 18 minutes at 500 bar until a temperature of 60 °C was achieved. The pre-warmed (60 °C) dextran solution was added to the iron oxide suspension and the mixture was circulated at 1000 bar until a temperature of 80 °C was achieved. Circulation was continued at 1000 bar and 80 °C for 20 minutes, and after stopping, the suspension was transferred to a 500 ml glass flask and was allowed to cool to room temperature. When the suspension had cooled, the particles were washed by magnetic separation in a high gradient magnetic field column (QuadroMACS with LD columns, Miltenyi Biotec GmbH, Bergisch-Gladbach, Germany) with 5 ml water per column. The magnetic column was removed from the magnet and the dextran coated magnetic particles were eluted with 2 ml of water per column. The HGMF wash was repeated until the suspension was completely recovered. The suspension was filtered using 0.22 µm PES (polyethersulfone) filter (Carl Roth GmbH, Karlsruhe, Germany).

For carboxymethyl-dextran coating, 2 g CM-carboxymethyl sodium salt (SIGMA-ALDRICH Chemie GmbH, Taufkirchen, Germany) was dissolved in 15 ml water at room temperature. 5 ml of citrate MNP suspension (50 mg/ml) was added and the mixture was stirred for 1 hour at 100 °C (120 rpm). The particles were washed as described above, and the washed suspension was filtered using glass fiber filter (Millex®-AP, Carl Roth GmbH, Karlsruhe, Germany).

For PEG-PEI coating, 2 ml of PEG-PEI (37% aqueous solution, M.W. 50,000; SIGMA-ALDRICH Chemie GmbH, Taufkirchen, Germany) was dissolved in 3 ml water. 5 ml of citrate MNP suspension (50 mg/ml) was added and the mixture was shaken for 16 hours at room temperature. Particles were washed and filtered as described for CM-dextran coated particles above.

For methoxy-PEG-phosphate + rutin coating, 530 µl of methoxy-PEG 5000 phosphate (750 mg/ml; SIGMA-ALDRICH Chemie GmbH, Taufkirchen, Germany) was added to 10 ml of citrate stabilized MNP suspension (50 mg/ml). Next, 100 mg rutin hydrate (SIGMA-ALDRICH Chemie GmbH, Taufkirchen, Germany) was dissolved in 5 ml methanol and added to the nanoparticle mixture. The mixture was stirred for 30 minutes at 50 °C, and methanol was removed by vacuum using a Laborota 4011 (Heidolph Instruments GmbH, Schwabach, Germany). The particles were centrifuged at 10,000 rpm for 10 minutes. Filtration of the supernatant was performed using a 0.22 µm PES filter.

### MNP size measurement

The mean hydrodynamic diameters of the MNPs was measured by dynamic light scattering (Zetasizer Nano-ZS90; Malvern Instruments Limited, Worcester, U.K.) at an iron concentration of c(Fe) = 0.4 mg/ml in water. The mean particle diameter Z(Avg) is given as result of the cumulative analysis of the autocorrelation function. The polydispersity index P.I. is a measure of the quality of the size distribution. A ‘monodisperse’ suspension has a polydispersity index <0.25.

### Zeta potential with pH

For all zeta potential measurements, the particles were suspended in 12 ml water at an iron concentration of 0.4 mg/ml. 0.25 M NaOH was added to pH = 10. Then 0.25 M HCl was added to measure the zeta potential in the pH range of 10 to 2.

### Magnetic susceptibility

The dynamic magnetic susceptibility of the particles was measured at c(Fe) = 1 mg/ml with a dynamic susceptometer (DynoMag, RISE Acreo, Gothenburg, Sweden) in a frequency range of 6 s^−1^ to 2.50 × 10^5^ s^−1^. The applied magnetic field during measurement was <400 A/m. The sample concentration of 1 mg/ml for each type of coated MNPs was achieved by diluting the stock samples for which iron concentrations were measured (Table 1). Thus, an additional uncertainty of ± 5% in the final sample concentration (1 mg/ml) was present due to sample preparation.

### Stability in PBS

Physiological conditions were simulated with phosphate buffered saline (PBS) at pH 7.4. The particle size distribution was compared by PCS measurement of the coated MNPs in water and in 0.01 M PBS buffer (SIGMA, P3813, pH = 7.4).

### Iron content measurement

The iron concentration of the MNPs was measured after decomposition of the iron oxide with concentrated hydrochloric acid, followed by spectrophotometric measurement with the Spectroquant® -kit (Merck) against a Titrisol®-iron standard (Merck). The solid content of each suspension was determined by gravimetry. Therefore 3 × 100 µl of particle suspensions were transferred to a weighing dish with known mass and dried for 30 min at 70 °C. After cooling to room temperature the mass of the dishes was determined. The solid concentration of the suspensions was calculated from the mass difference of the weighing dishes before and after particle loading.

### Specific loss power (SLP) measurement

SLP was evaluated for coated and uncoated MNPs in the field range of 8 kA/m to 54 kA/m (peak-to-peak) and at fixed frequency of 1.5 × 10^5^ s^−1^ ± 1.0 × 10^4^ s^−1^. The alternating magnetic field (AMF) heating system, sample preparation and SLP evaluation have been described in detail elsewhere^50–53^. In brief, an 80 kW induction heating system (PPECO, Watsonville, CA) was used as the power supply. The solenoid coil (16 cm length) with four turns, formed using cylindrical sections of copper plate, was constructed such that the entire sample volume was exposed to a homogenous (±<10%) magnetic field in a ~125 cm^3^ volume. At an applied voltage of 650 V at 1.4 × 10^5^ s^−1^, a peak field amplitude of 47.9 ± 0.2 kA/m was measured (AMF Life Systems, Inc field probe) and uniformity over a length of 6.6 ± 0.3 cm was measured^52^. The calorimeter consisted of an insulating sample holder placed within the solenoid induction coil^51^. The magnetic field amplitude at ~1.5 ×  × 10^5^ s^−1^ (±10 × 10^5^ s^−1^) was fixed in the range of 8-54 kA/m by changing the power supply voltage. Assuming that the system is closed, i.e. no energy or mass exchange with environment occurs; and, the work done on/by the system is solely of a magnetic nature, then the loss power can be estimated from measurements of temperature change within the sample^51,53^. The specific loss power, SLP, is defined as the measured thermal loss power normalized by mass of magnetic material (Fe for iron oxide MNPs) and expressed in W/g Fe units and is calculated using the expression,

4 $${\rm{S}}{\rm{L}}{\rm{P}}{\rm{=}}({\rm{C}}{\rm{/}}{{\rm{m}}}_{{\rm{F}}{\rm{e}}}){\rm{\ast }}({\rm{\Delta }}{\rm{T}}/{\rm{\Delta }}{\rm{t}})$$

where *T* is temperature in °C, *t* is time in s, mFe is the equivalent mass of iron in g and *C* is the sample specificheat capacity in J/°C. This formula is only valid if the above stated assumptions hold. In experimental systems, the adiabatic criterion is never established throughout, thus compelling an analysis of the data to estimate SLP from portions of time-temperature data conforming to (quasi-)adiabatic conditions^51,53^.

For the SLP measurement, a 1 g nanoparticle suspension in deionized water (18 MΩ.cm) was placed in standard 5 ml polystyrene tubes and inserted into an insulating sample holder. Fiber-optic temperature probes and an optical conditioner (FISO Technologies Ltd., Quebec, Canada) were used to measure temperature *in situ*. Before commencing the measurements, each nanoparticle sample was allowed to equilibrate with the environment temperature to ensure it was constant with a maximum deviation of ± 0.02 °C in 10 s. Temperatures were measured every 0.6 s. The AMF power supply was turned on and temperature was monitored for 60 s or until a maximum temperature of 50 °C, whichever was achieved first. Water blanks were used at each field setting to correct for the calorimeter heat capacity. From the temperature vs time plots, mean SLP and variances inherent in the measurement were calculated using Equation 1 and by identifying all time ranges in a single heating experiment that satisfied quasi-adiabatic conditions^53^.

### *In vivo* MNP uptake

Animals were housed in an Association for Assessment and Accreditation of Laboratory Animal Care-accredited facility in compliance with the *Guide for the care and use of laboratory animals*^54^. All procedures were approved by the Johns Hopkins Institutional Animal Care and Use Committee. Twenty male athymic nude mice, aged 4-5 weeks and mean weight 24 grams, were obtained from Harlan (Frederick, MD). Mice were divided into 5 groups with 4 mice in each, and each mouse received injections of MNPs at a dose of 2 mg Fe/animal into retro-orbital (RO) plexus. Blood samples were collected from the RO sinus of each mouse 24 hours and 48 hours later. At 48 hours, mice were sacrificed and lungs, livers, and spleens were collected. For follow up histopathology studies, intravenous injections of MNPs were via tail vein. All collected tissues were lyophilized and prepared for ICP-MS using previously described procedures^55^.

### ICP-mass spectrometry

Seronorm™ Trace Elements Serum (SERO AS, Billingstad, Norway) was the standard reference material (SRM) used for analysis of blood and tissue samples. Samples and SRMs were weighed and quantitatively transferred to 7 ml Teflon microwave digestion vessels (Savillex Corporation, Eden Prairie, MN). 1 ml of Optima grade (67–69%) HNO_3_ (Fischer Scientific, Pittsburgh, PA) was added to each sample and to sample preparation blanks, and the samples were placed into 55 ml Teflon digestion vessels (CEM corporation, Matthews, NC) which were sealed after adding 10 ml of ultra-pure (18 MΩ·cm) water (Millipore Corporation, Billerica, MA) and placed into a microwave (Mars 5 Xpress, CEM Corporation, Matthews, NC) for digestion. Tissue samples were digested using single stage ramp-to-temperature method (15-minute ramp to 130 °C, with a hold of 10 minutes). Upon cooling, samples were diluted to 5% in polystyrene test tubes (Sarstedt, Nümbrecht, Germany) and an internal standard of Scandium was added to a concentration of 0.05 μg/ml. Analyses of diluted sample digests were carried out using inductively-coupled plasma mass spectroscopy (Agilent 7500ce, Agilent Technologies, Columbia, MD). For every batch of 20 tissue samples, three samples of Seronorm whole blood and four reagent blanks were digested and analyzed. Each measurement was blank-corrected using the average iron value in the reagent blanks, multiplied by the dilution factor, and adjusted based upon the recovery of Fe from whole blood. A 10-point calibration curve (0, 1, 5, 10, 50, 100, 500, 1000, 5000 and 10,000 µg/l) was obtained and the iron mass (μg) was calculated per gram of tissue sample.

#### Histology and Immunohistochemistry

Lungs collected from animals after 24 or 48 hours were fixed in 10% formalin for 48 hours at room temperature. They were then paraffin embedded and sectioned for hematoxylin and eosin (H&E) staining to visualize cellular morphology. Adjacent sections were prepared for Prussian blue staining to evaluate the iron distribution. The slides were digitally scanned using an Aperio ScanScope At system (Aperio, Vista CA) at 20× (H&E) or 40× (Prussian blue) magnification. Immunohistochemistry analysis of co-localization of nanoparticles with macrophages and monocytes was performed using anti-ionized calcium-binding adapter molecule 1 (anti-IBA-1) antibody. IBA-1 is a pan-macrophage marker that also labels some other myeloid cells including subpopulations of dendritic cells^56^. Tissue sections were stained with IBA-1 antibody on positively charged slides that were deparaffinized on a heating block followed by washing in 2 changes of xylene. The slides were then dehydrated in 100%, 95% and 70% ethanol followed by water. The slides were steamed for 45 minutes in EDTA buffer for antigen retrieval and then incubated with anti-IBA-1 antibody (1:2500- Wako 019-19741) for 45 minutes at room temperature. After washing unbounded antibody, the sections were incubated with secondary antibody for 30 minutes at room temperature (PowerVision Poly-HRP anti-Rabbit IHC Detection Systems Novocastra, Leica Biosystems, Buffalo Grove, IL). After washing, the slides were developed with DAB reagent (DAB fast –Sigma Aldrich, St.Louis, MO) for 20 minutes and counter stained with hematoxylin at room temperature. The slides were visualized and photographed using EVOS imaging system at 40× magnification (Thermo Fisher Scientific, Waltham, MA USA).

### Data availability

Data obtained during the course of this study are available upon request.

## Electronic supplementary material

Supplementary Materials
