# Supplementary material for: Physical characterization and in vivo organ distribution of coated iron oxide nanoparticles
Source: Sci Rep. 2018 Mar 20;8:4916. doi: 10.1038/s41598-018-23317-2 (PMC5861066; doi:10.1038/s41598-018-23317-2)
Supplement: Supplementary file 1 — Supplementary Materials [file 41598_2018_23317_MOESM1_ESM.pdf]

## Supplementary information

## Physical characterization and *in vivo* organ distribution of coated iron oxide nanoparticles

*Anirudh Sharma, Christine Cornejo, Jana Mihalic, Alison Geyh, David E. Bordelon, Preethi Korangath, Fritz Westphal, Cordula Gruettner, and Robert Ivkov*

### Theoretical Néel relaxation time calculation

$$\tau_N = \tau_A \left(\frac{\sqrt{\pi}}{2}\right)\left(\sqrt{\frac{k_B T}{K V_M}}\right) e^{K V_M / k_B T}$$

Attempt frequency. $\tau_A = 10^{-9}$ s

The magnetic volume, $V_M = = \frac{4}{3} * \pi * (18 * 10^{-9})^3$ , assuming a spherical magnetic domain of 36 nm diameter.

Anisotropy constant, K = $0.366*10^4 J/m^3$.

Boltzmann constant $k_B = 1.38*10^{-23}$ $m^2.kg.s^{-1}K^{-1}$.

Temperature, T = 300 K.

Thus, substituting above values in the formula for Néel relaxation gives $\tau_{N} =$ 0.451 s.

The assumed single magnetic domain sphere having a diameter of 36 nm for the dextran coated MNP is incorrect, and instead data better complies with the more complex core-shell magnetic multi-domain model proposed for dextran coated MNPs in earlier work[19].

### ACS of immobilized particles:

MNP were embedded into 2% agarose. An agarose tablet (0.5 g, Carl Roth GmbH, Karlsruhe, Germany, HP67.1) was dissolved in 25 ml water within 5 min at room temperature. The agarose solution was heated in a water bath until reflux. After cooling to about 70-80 °C, 180 µl of the agarose solution were transferred to a 1 ml vial shell (VWR International, Darmstadt, Germany,

548-0042), which contained 20 µl of particle suspension with an iron concentration of 10 mg/ml. The gel was formed upon cooling to room temperature.

**Numerical Inversion of ACS Data**:

An analysis of ACS data by numerical inversion according to equation 3 was previously described by P Bender et al (The code for data processing was written in Python and was received directly from the author). In brief, a discrete distribution function for relaxation times was extracted by direct numerical inversion of the ACS data. A regularized inversion method (initially developed for the analysis of small angle scattering data) based on indirect Fourier analysis was adapted to Debye type relaxation processes. This model-independent approach enables the calculation of the relaxation time distribution p(τ) without *a priori* models for the distribution of the iron oxide core diameter and the internal magnetic interaction (relevant for internal relaxation) or the distribution of the hydrodynamic diameter (relevant for external relaxation).

The relaxation distribution p(τ) for dextran coated MNP shown in Figure S2 exhibits a broad distribution of relaxation times at $10^{-4}$ s related to Brownian relaxation. With $\omega\tau=1$, this corresponds to a frequency of 1.6 kHz and is in good agreement with the position of the imaginary peak of colloidal particles in Figure S1. The application of the model described by equation 1 results in the assignment of a hydrodynamic diameter of about 67 nm to this distribution mode, which is comparable to the DLS size of 77 nm. A second mode at $3 \times 10^{-6}$ s is significant and can be related to internal Néel relaxation[42]. The shadowed time ranges in the distribution indicate unreliable solutions due to the limitations in the frequency range of ACS. The maximum in the $10^{-7}$ s range cannot be further analyzed, but should be related to the

relaxation of single iron oxide cores with 15 nm in size [19]. The relaxation time distribution for immobilized MNP (red in Figure S2) is indifferent and nearly equally distributed within the time range observed.

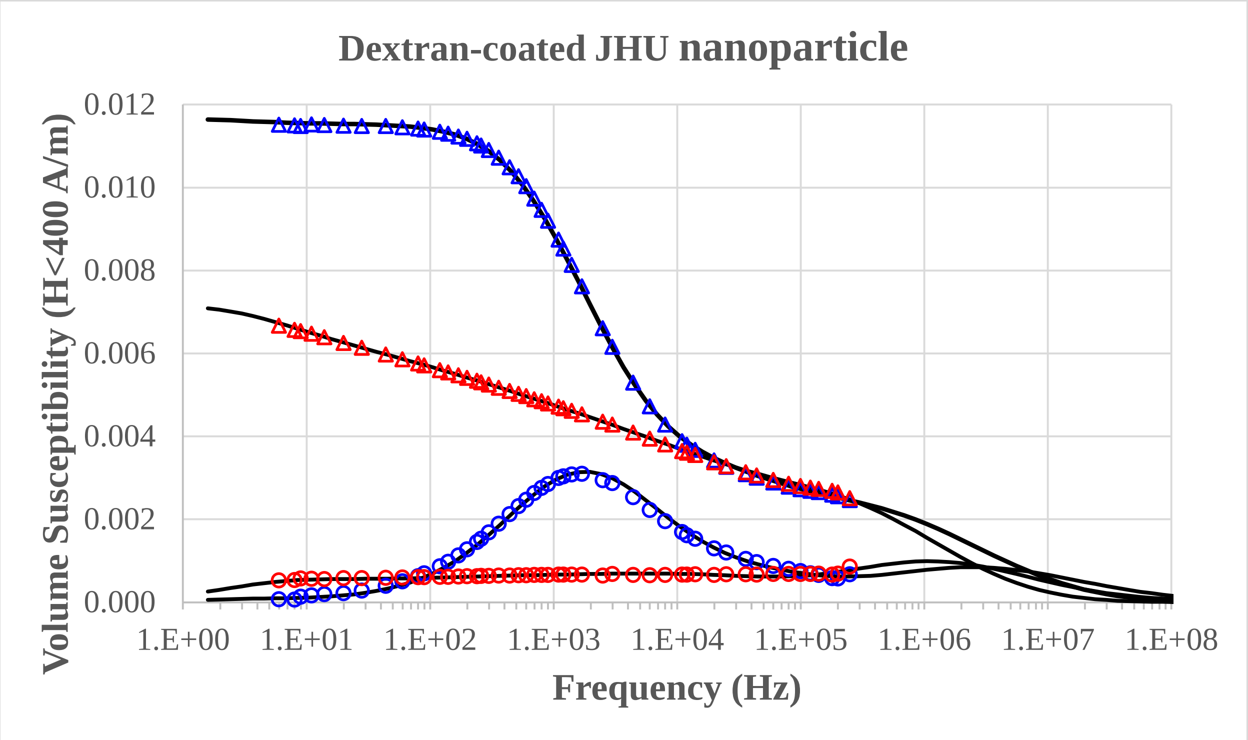


**Figure S1:** Volume susceptibility of dextran coated MNPs showing real (triangles) and imaginary components (circles) with frequency of applied external (AC) magnetic field. For the comparison with colloidal data (blue), the AC-susceptibility of immobilized particles is shown (red) for comparison with free particles (blue). Solid lines represent the fit results after numerical inversion.

**Figure S1**

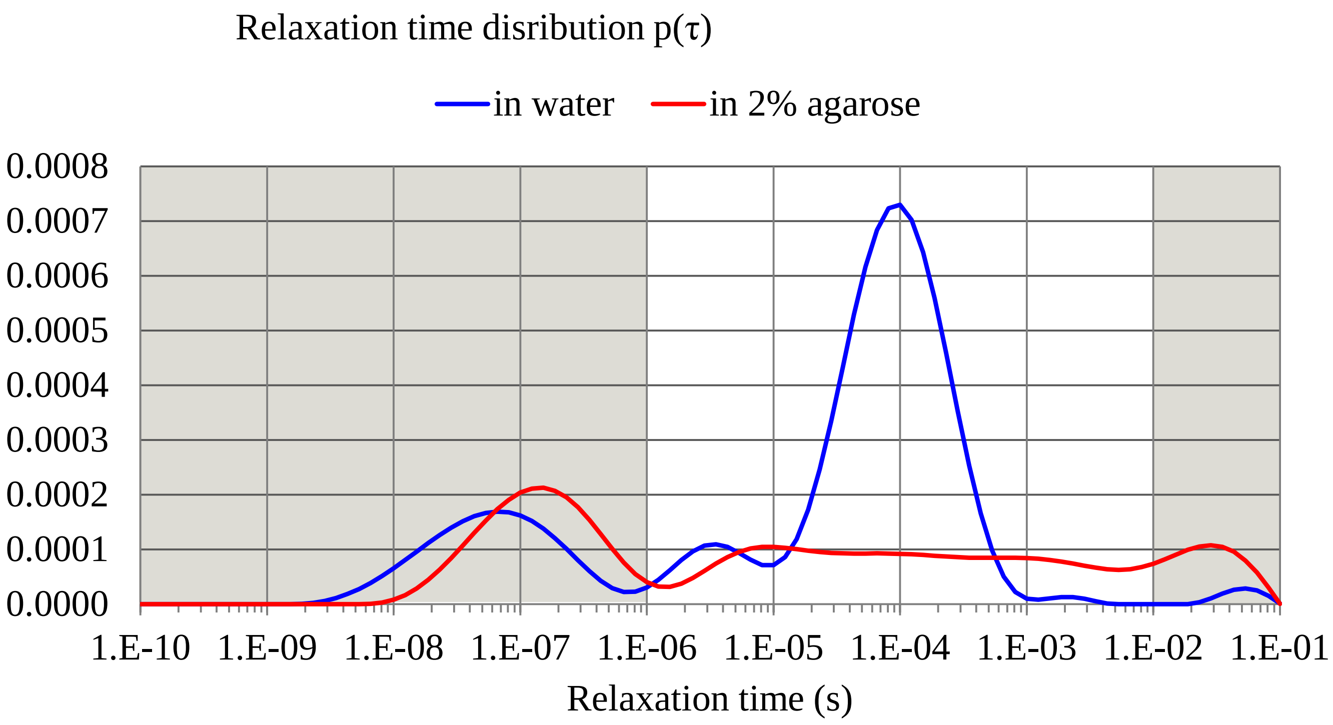


**Figure S2:** Relaxation time distribution p(τ) for dextran coated MNP calculated from measured data shown in Figure S1.

**Figure S2**

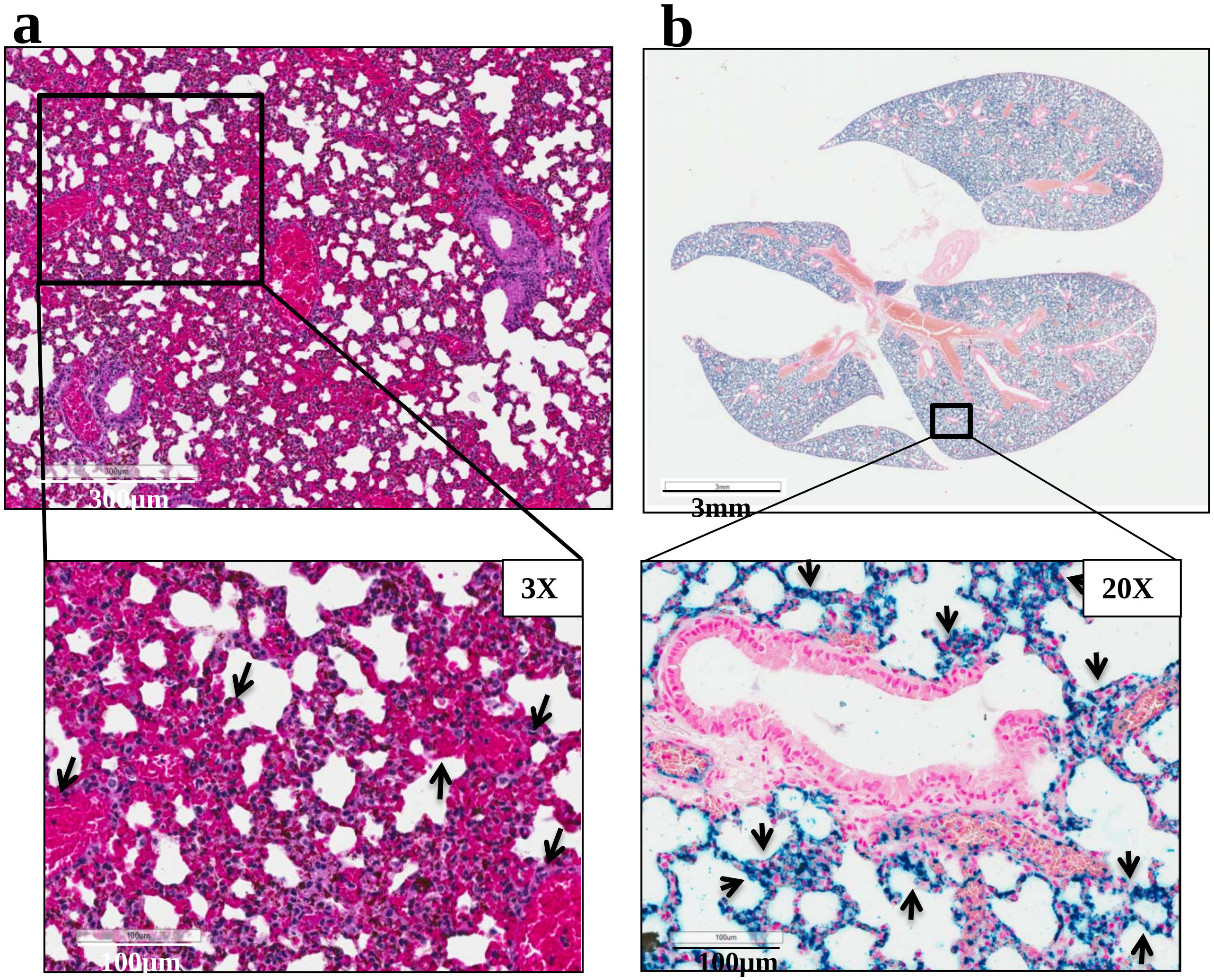


**Figure S3:** Representative images of lung sections of animals injected with PEG-PEI MNPs (2 mg Fe) showing signs of acute toxicity (a) H&E stained sections show widespread vascular disruption (black arrows). (b) Prussian blue staining shows blue coloration due to nanoparticle accumulation and disrupted red blood cells (black arrows). Lower panels show sections indicated by boxed regions at higher magnification.

**Figure S3**

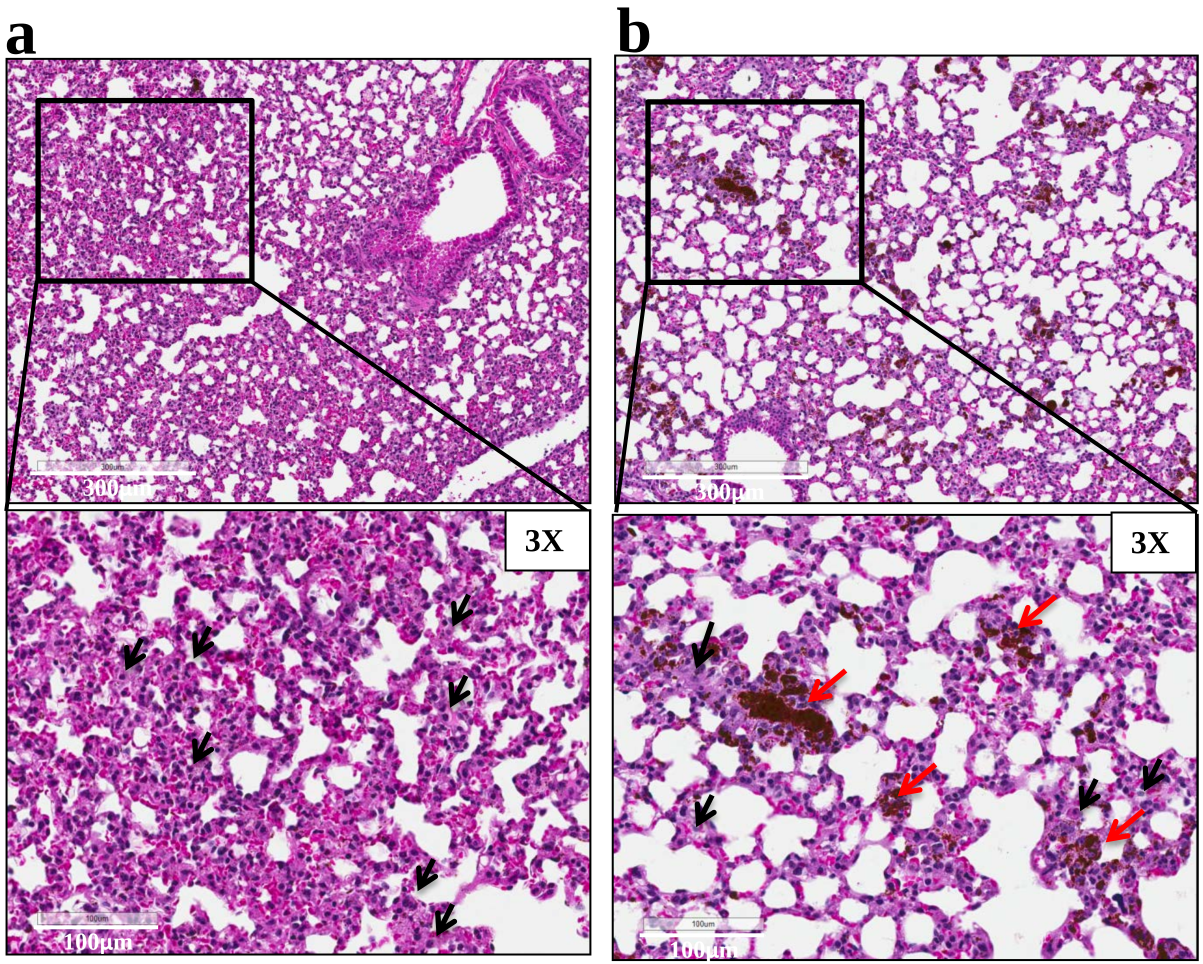


**Figure S4:** Representative images of H&E stained lung sections of mice injected with (a) PEG-PEI MNPs (1mg Fe) showing congested alveolar epithelium with immune cell infiltration (black arrows) (b) CM-Dextran coated MNPs showing clustered particles (red arrows) within interstitial spaces with less immune cell infiltration than that of PEG-PEI MNP injected animals. As in Figure S3, lower panels show boxed regions at higher magnification.

**Figure S4**

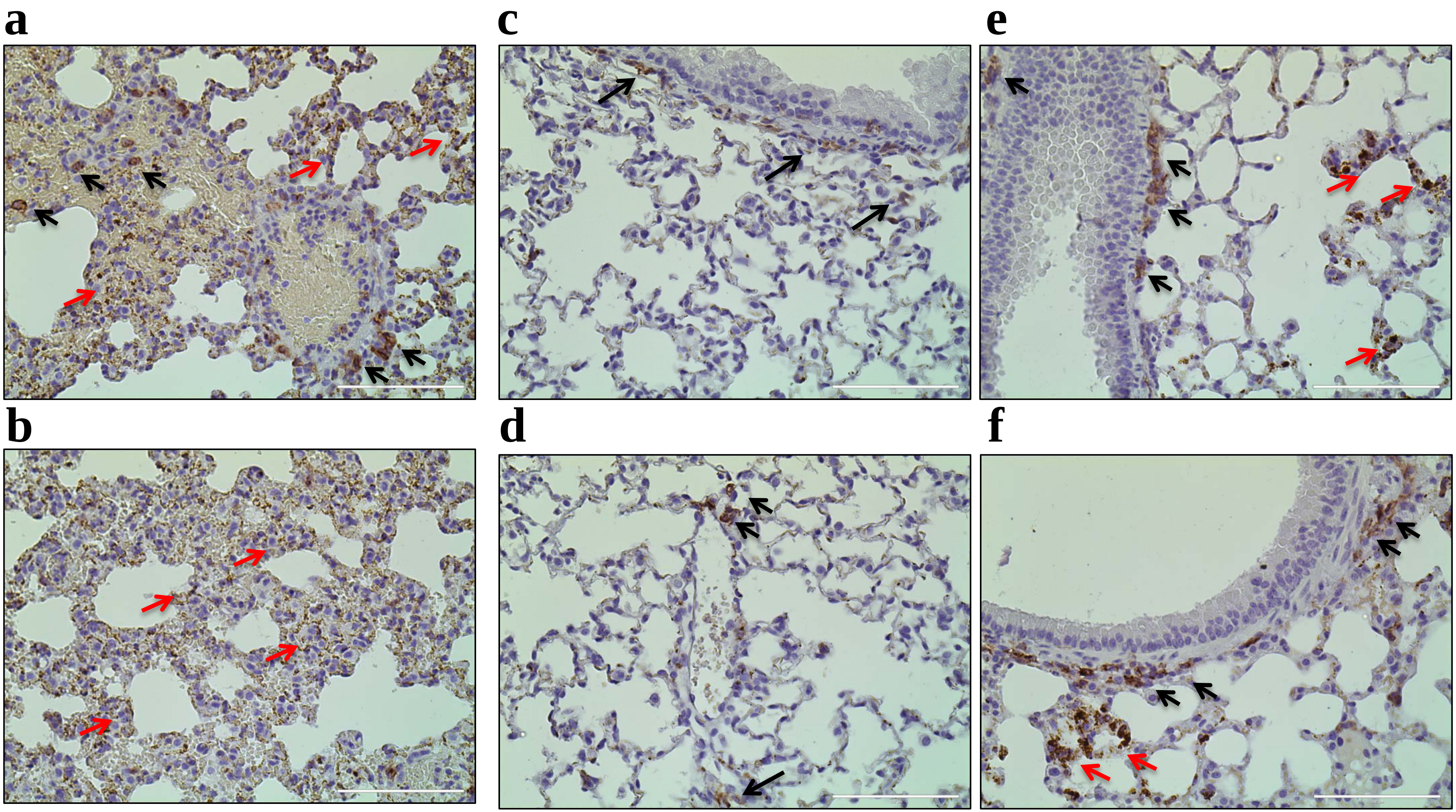


**Figure S5:** Representative images of IBA-1 stained lung sections showing no clear co-localization of MNPs with IBA-1 positive cells. (a&b) IBA-1 staining of lungs from animal injected with PEG-PEI MNPs (2 mg Fe) showing few IBA-1 positive cells (black arrows). Non-specific color reaction of MNPs revealed as brown coloration with H&E throughout the section (red arrow). (c&d) Few IBA-1 positive cells are seen in animals injected with PEG-PEI MNPs (1 mg Fe) and no evidence of co-localization with MNPs. (e&f) Similar observations were made in lung sections from CM-Dextran MNP injected animals (red arrows indicate non-specific staining).

**Figure S5**
